# Pro-inflammatory markers predict response to sequential pharmacotherapy in major depressive disorder: a CAN-BIND-1 report

**DOI:** 10.1192/j.eurpsy.2023.659

**Published:** 2023-07-19

**Authors:** M. I. Husain, J. A. Foster, B. L. Mason, S. Chen, W. Wang, S. Rotzinger, S. Rizvi, K. Ho, R. Lam, G. MacQueen, R. Milev, B. N. Frey, D. Mueller, G. Turecki, M. Jha, M. Trivedi, S. H. Kennedy

**Affiliations:** ^1^ Campbell Family Mental Health Research Institute, Centre for Addiction and Mental Health; ^2^Psychiatry, University of Toronto, Toronto, Canada; ^3^UT Southwestern, Houston, United States; ^4^Unity Health, Toronto; ^5^Psychiatry, University of British Columbia, Vancouver; ^6^Psychiatry, University of Calgary, Calgary; ^7^Psychiatry, Queen’s University, Kingston; ^8^McMaster University, Hamilton; ^9^Psychiatry, McGill University, Montreal, Canada

## Abstract

**Introduction:**

Despite replicated cross-sectional evidence of aberrant levels of peripheral inflammatory markers in individuals with major depressive disorder (MDD), there is limited literature on associations between inflammatory tone and response to sequential pharmacotherapies.

**Objectives:**

To assess associations between plasma levels of pro-inflammatory markers and treatment response to escitalopram and adjunctive aripiprazole in adults with MDD.

**Methods:**

In a 16-week open-label clinical trial, 211 participants with MDD were treated with escitalopram 10– 20 mg daily for 8 weeks. Responders continued on escitalopram while non-responders received adjunctive aripiprazole 2–10 mg daily for 8 weeks. Plasma levels of pro-inflammatory markers – C-reactive protein, Interleukin (IL)-1β, IL-6, IL-17, Interferon gamma (IFN)-Γ, Tumour Necrosis Factor (TNF)-α, and Chemokine C–C motif ligand-2 (CCL-2) - measured at baseline, and after 2, 8 and 16 weeks were included in logistic regression analyses to assess associations between inflammatory markers and treatment response.

**Results:**

Pre-treatment levels of IFN-Γ and CCL-2 were significantly higher in escitalopram non-responders compared to responders. Pre-treatment IFN-Γ and CCL-2 levels were significantly associated with a lower of odds of response to escitalopram at 8 weeks. Increases in CCL-2 levels from weeks 8 to 16 in escitalopram non-responders were significantly associated with higher odds of non-response to adjunctive aripiprazole at week 16.

**Conclusions:**

Pre-treatment levels of IFN-Γ and CCL-2 were predictive of response to escitalopram. Increasing levels of these pro-inflammatory markers may predict non-response to adjunctive aripiprazole. These findings require validation in independent clinical populations.

**Disclosure of Interest:**

None Declared

